# Barnacle inspired high-strength hydrogel for adhesive

**DOI:** 10.3389/fbioe.2023.1183799

**Published:** 2023-04-03

**Authors:** Dezhao Hao, Xingchao Li, Enfeng Yang, Ye Tian, Lei Jiang

**Affiliations:** ^1^ CAS Key Laboratory of Bio-inspired Materials and Interfacial Science, Technical Institute of Physics and Chemistry, Chinese Academy of Sciences, Beijing, China; ^2^ School of Future Technology, University of Chinese Academy of Sciences, Beijing, China; ^3^ Beijing Institute of Future Science and Technology on Bioinspired Interface, Beijing, China

**Keywords:** barnacle, hydrogel, bio-inspired, phase separation, under water and oil adhesive

## Abstract

Barnacle exhibits high adhesion strength underwater for its glue with coupled adhesion mechanisms, including hydrogen bonding, electrostatic force, and hydrophobic interaction. Inspired by such adhesion mechanism, we designed and constructed a hydrophobic phase separation hydrogel induced by the electrostatic and hydrogen bond interaction assembly of PEI and PMAA. By coupling the effect of hydrogen bond, electrostatic force and hydrophobic interaction, our gel materials show an ultrahigh mechanical strength, which is up to 2.66 ± 0.18 MPa. Also, benefit from the coupled adhesion forces, as well as the ability to destroy the interface water layer, the adhesion strength on the polar materials can be up to 1.99 ± 0.11 MPa underwater, while that of the adhesion strength is about 2.70 ± 0.21 MPa under silicon oil. This work provides a deeper understanding of the underwater adhesion principle of barnacle glue. Furthermore, our bioinspired strategy would provide an inspiration for the fabrication of high mechanical gel materials, and the rapid strong adhesive used in both water and organic solvents.

## Introduction

Barnacle, a very common marine organism, has a very strong underwater adhesion ability. It can cause serious surface damage to many natural and artificial materials in the sea (Aldred and Clare, 2008; Murosaki et al., 2011; Kamino, 2013), and even can firmly adhere to the skin or shell of some animals (Pugliese et al., 2012). Barnacle cement is considered to be the most durable and hardest connection among aquatic organisms (Abbott, 1990). Adhesive is a protein material (>90% protein), while the rest is composed of carbohydrates, ash and trace lipids (Walker, 1999). The barnacle adhesion protein can rapidly self-assemble in water and adhere the barnacle itself to various substrates (Cheung et al., 1977; Wiegemann and Watermann, 2003). Among barnacle adhesive proteins, there are only common amino acids, and there is no special adhesive chemical like L-dopa in the mussel (Wilker, 2011; Kord Forooshani and Lee, 2017). The hydrogen bond and ionic bond in barnacle cement are considered to be weak and cannot realize the main cross-linking mechanism. Due to the resistance of the adhesive to thioglycolate, disulfide bonds are also excluded (Naldrett, 1993). On the contrary, researchers (Barnes and Blackstock, 1976) showed that the anionic detergent sodium dodecyl sulfate (SDS) containing the reducing agent 2-mercaptoethanol was sufficient to dissolve the cement, which led to the hypothesis that hydrophobic interaction and sulfur crosslinking were the key components of the cement matrix. Other evidence shows that the high hydrophobicity of barnacle proteins is the main reason for the insolubility of these proteins (Naldrett and Kaplan, 1997).

However, the key problem to achieve effective adhesion underwater is how to break through the water layer on the solid surface (Waite, 1987; Maier et al., 2015). The water film will prevent the effective contact between various adhesives and the surface, and it will also occupy a certain volume and is difficult to be discharged after the adhesive is cured (Shao and Stewart, 2010; Akdogan et al., 2014; Zhao et al., 2016; Zhao et al., 2017). Therefore, it is very important to study the competitive forces between adhesive molecules and interfacial water on the surface (Narayanan et al., 2021). At present, the strategies of underwater and organic solvent adhesion are mainly divided into the following categories: 1) underwater adhesion is achieved by using stronger hydrogen bonds, such as L-dopa or dopamine based adhesives (Ryu et al., 2011; Wang et al., 2015; Cui et al., 2019) and Phragmatopoma californica (Zhao et al., 2005). But the adhesion strength that can be achieved is relatively low; 2) The adhesion between water and organic solvent is realized by solvent exchange (Zhao et al., 2016; Xu et al., 2020; Wan et al., 2023). The adhesion strength is still very limited; 3) Underwater adhesion can be achieved by hydrophobic interaction, such as PDMS tape (Wan et al., 2019) and organogel coating (Li et al., 2020); 4) It is still difficult to achieve high adhesion strength by extruding the water layer with special structures, such as the suction cups of octopi (Baik et al., 2017), clingfish (Ping et al., 2018), etc. Compared with natural underwater adhesives, it is still a challenge to design and prepare an adhesive that has both the adhesion strength and the convenience under water and specially under organic solvents.

Here, we imitate the solidification and interface interaction principle of barnacle glue, and use two hydrophilic polymers PMAA and PEI to form a relatively hydrophobic polymer network through hydrogen bonding and electrostatic self-assembly hydrogel in aqueous solution. In this gel, there are hydrogen bond, electrostatic force and hydrophobic interaction, and these water-soluble monomers can diffuse and mix with the interface water to destroy the interface water layer. We plan to test the gel to carry out underwater and organic solvent adhesion tests in imitation of the underwater adhesion process of barnacles.

### Chemicals and materials

Polyethylene imine (PEI, 50% aqueous solution, molecular weight Mw 70000), methacrylic acid (MAA), Dimethyl silicone oil (viscosity 10 mPa s), ammonium persulfate, guanidine thiocyanate (GT), sodium dodecyl sulfate, sodium fluorescein, the above reagents were purchased from Macklin Biochemical Technology; Hydrochloric acid (36 wt%), sodium carbonate, sodium bicarbonate, ethyl acetate, ethanol, n-hexane, and the above reagents are purchased from Sinopharm chemicals; 1,1′-Dioctadecyl-3,3,3′,3′-tetramethylindocarbocyanine perchlorate (DiI, purchased form Sigma-Aldrich). The above reagents are directly used without purification in the experiment.

### Preparations of hydrogel

First, dilute 30 g of 50% PEI aqueous solution with 70 g of water, then add 30 g of methacrylic acid, then stir evenly and remove bubbles in it by ultrasonic for 10 min. During polymerization, add 0.3 g ammonium persulfate and quickly stir it evenly, then pour the mixed solution into the mold and wait for curing at room temperature. For other proportions of gel, please refer to Supplementary Table S1 in the Supplementary Material.

### Characterizations of hydrogel structures and wettability

Before testing SEM, hydrogel samples need to be freeze-dried first, then sprayed with gold, and then put into an electron microscope (Hitachi, SU8010) to observe its porous structure. The voltage is 10 kV, and the magnification is 200-10000 times.

The surface wettability of gel samples was tested by the contact angle tester OCA-20 machine (Dataphysics Germany). Use 2 μL of pure water for each test. Change different positions after each test, take 5 data values from each sample and average them.

The water-soluble fluorescein sodium and oil-soluble DiI water and n-hexane were prepared into 10 nM solution respectively, and then the samples were put into them for dyeing, and then the dyed samples were put into Nikon Confocal Microscopes C2 system for observation.

### Mechanical tests

The sample for mechanical test is prepared with a PTFE mold, as shown in Supplementary Figure S1. The width of the test part is 6 mm, the thickness is 2 mm, and the cross-sectional area is 12 mm^2^. The model of tension machine is CTM2100/1KN (Xie Qiang Instrument Manufacturing (Shanghai) Co., Ltd.), the test range of the sensor is 0-1000 N.

### Adhesive properties

Adhesion test samples use the basement sized 1 cm × 2.5 cm. Place one adhesive substrate in water or organic solvent, and then add pre-gel solution 500 μL. Then, another adhesive substrate is pressed on the adhesive substrate with hydrogel pre-gel solution in water or organic solvent, and the included angle between the two substrates is kept at 90° until the hydrogel is cured. The adhesion area of all adhesive samples obtained is 1 cm^2^.

## Results and discussion

### Chemical structure design and self-assembly mechanism

**FIGURE 1:**
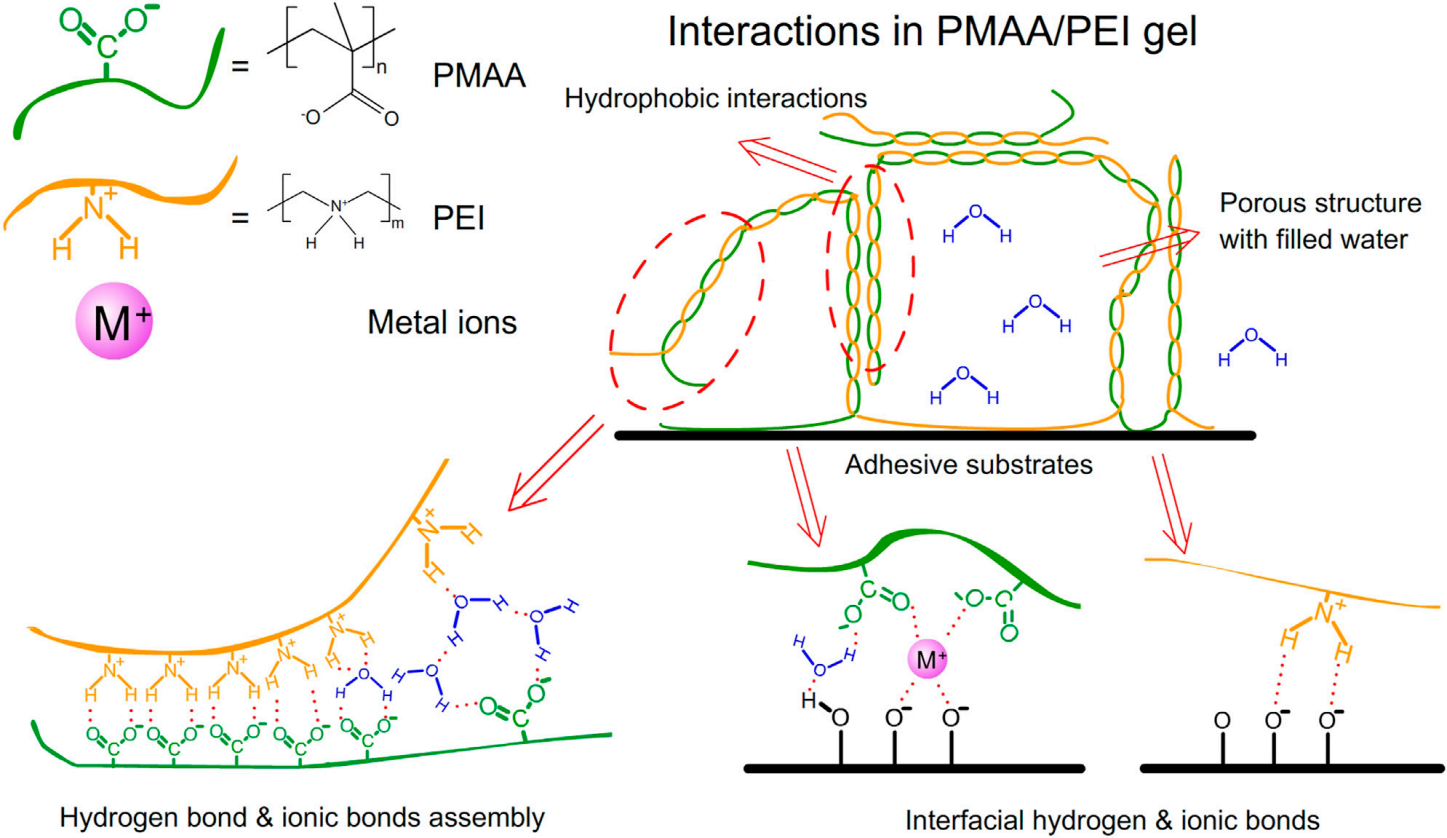
Interactions in PMAA/PEI gel.

**FIGURE 2:**
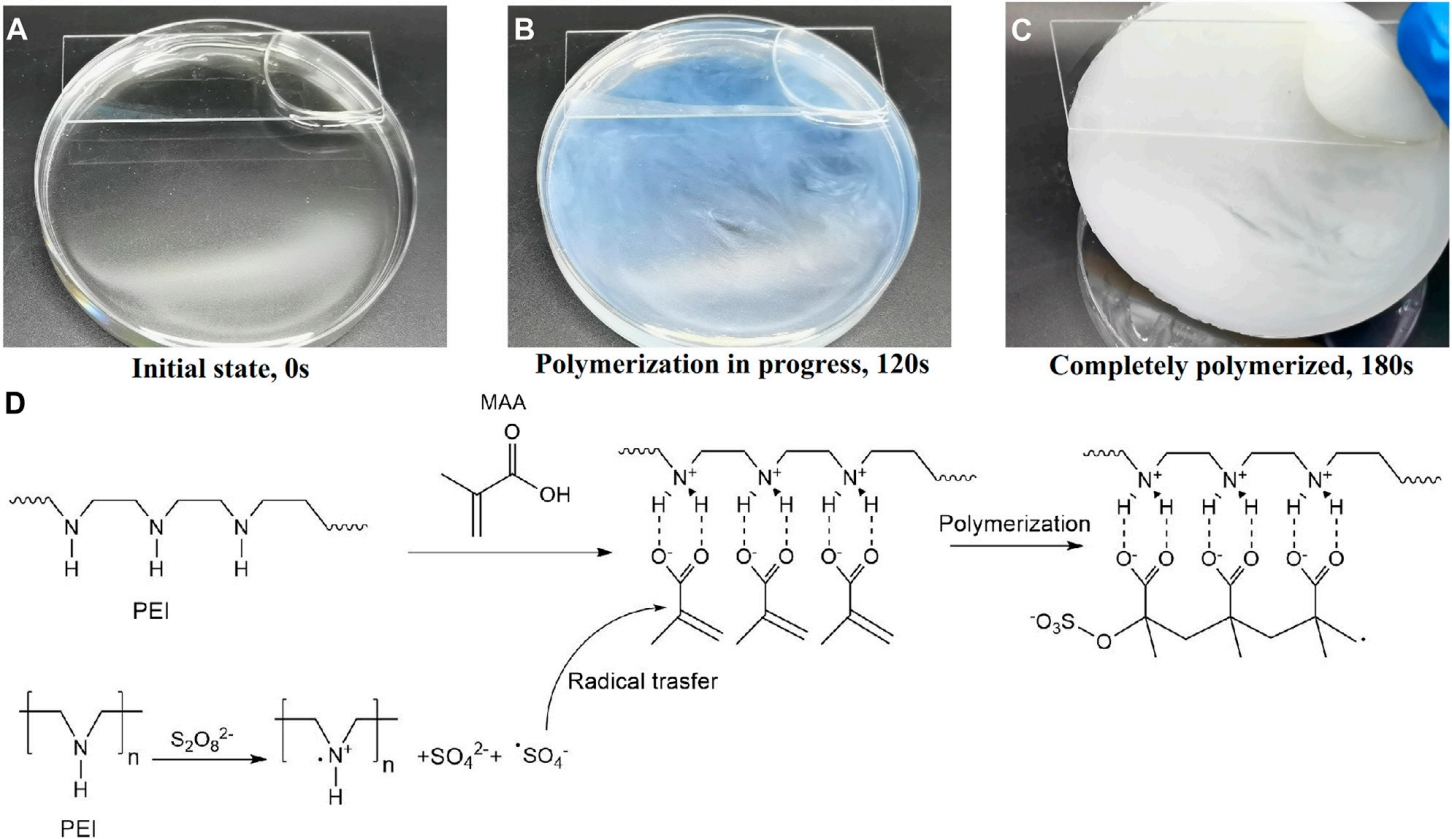
The polymerization process of PMAA/PEI gel. **(A–C)** The photograph of the polymerization progress. **(D)** The principle of room temperature thermal polymerization.

Barnacle glue contains a lot of charged amino acids and hydrophobic components, which can be self-assembled by electrostatic and hydrophobic interactions to form barnacle glue with underwater adhesion (Kamino et al., 2000; Kamino, 2001; Yan et al., 2020). Inspired by barnacle, we designed a hydrogel with a large number of amino and carboxyl groups, which contains the polyethyleneimine (PEI) and the monomer methacrylic acid (MAA). The polymerization is initiated by ammonium persulfate (APS). After polymerization, these two polymers can form high-strength PMAA/PEI self-assembled hydrogel (hereinafter referred to as PMAA/PEI gel, as shown in Figure 1) by electrostatic and hydrogen bonding. Additionally, when the molar ratio of the two polymer units is close to 1:1, MAA and PEI in PMAA/PEI gel can form a self-assembly structure relying on the internal hydrogen bond and ionic bond and can shield the charge, so that the electrostatic interaction and external hydrogen bond interaction are weakened and the assembly becomes hydrophobic, and then the hydrophobic interaction continues to assemble to obtain a phase separation structure. Figures 2A–C shows the polymerization process of gel. It can be seen that gel is polymerized by clear and transparent solution at room temperature, and can be completely solidified into white and hard gel in only about 180 s. Figure 2D explains the polymerization principle of gel. On the one hand, PEI can act as an accelerator of persulfate, which can form a large number of free radicals to promote polymerization when reacting with persulfate at room temperature; On the other hand, PEI is positively charged in solution, which will rely to electrostatic force to attract negatively charged methacrylic acid anions, so it is very conducive to the rapid polymerization of methacrylic acid and the formation of assembly.

**FIGURE 3:**
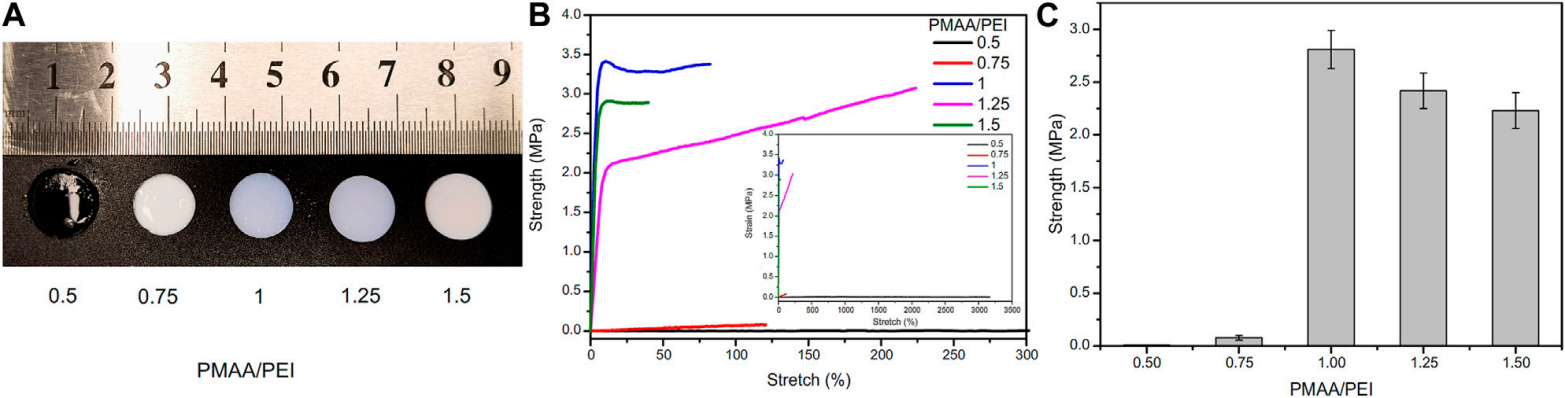
Hydrogel samples at different PMAA/PEI molar ratios. **(A)** Photos of these samples, of which PMAA/PEI = 0.5 is still transparent, while other samples are white, indicating that these samples have shown relatively obvious phase separation; **(B)** Elongation curves of several samples, the illustration of which is a complete force curve, in which the elongation at break of the sample with PMAA. PEI = 0.5 exceeds 3,000%; **(C)** The breaking strength of gel with several ratios, of which the gel with PMAA/PEI molar ratio of 1:1 has the best performance, and this ratio is used by default in subsequent experiments.

PMAA/PEI gel contains no chemical cross-linker, but only relies on the electrostatic, hydrogen bond and hydrophobic interaction between the two polymers to form gel. Therefore, the final properties of gel are very related to the proportion of the two substances. Figure 3A and Supplementary Video S1 shows the photos of PMAA/PEI gel with different polymer unit mole ratios (The ratio of amino groups in PEI to carboxyl groups in PMAA). When PMAA: PEI = 0.5, the gel network is positively charged as a whole. Due to the good hydrophilicity of PEI, the gel remains transparent and has low strength, but has an elongation at break of more than 3,000%, as illustrated in Figure 3B. When PMAA: PEI = 1, the two polymers in the gel can just be assembled 1:1, and the strength of the gel also reaches the highest, as shown in Figure 3C. When the proportion of PMAA is further increased, the gel is acidic in the case of excessive PMAA. The hydrophilicity of PMAA will decline, so the gel can still keep a good hydrophobic assembly phase separation state, and the strength will only decrease slightly. Based on the above experimental results, PMAA: PEI = 1 gel was used as the research object in all subsequent studies.

### Wettability study

**FIGURE 4:**
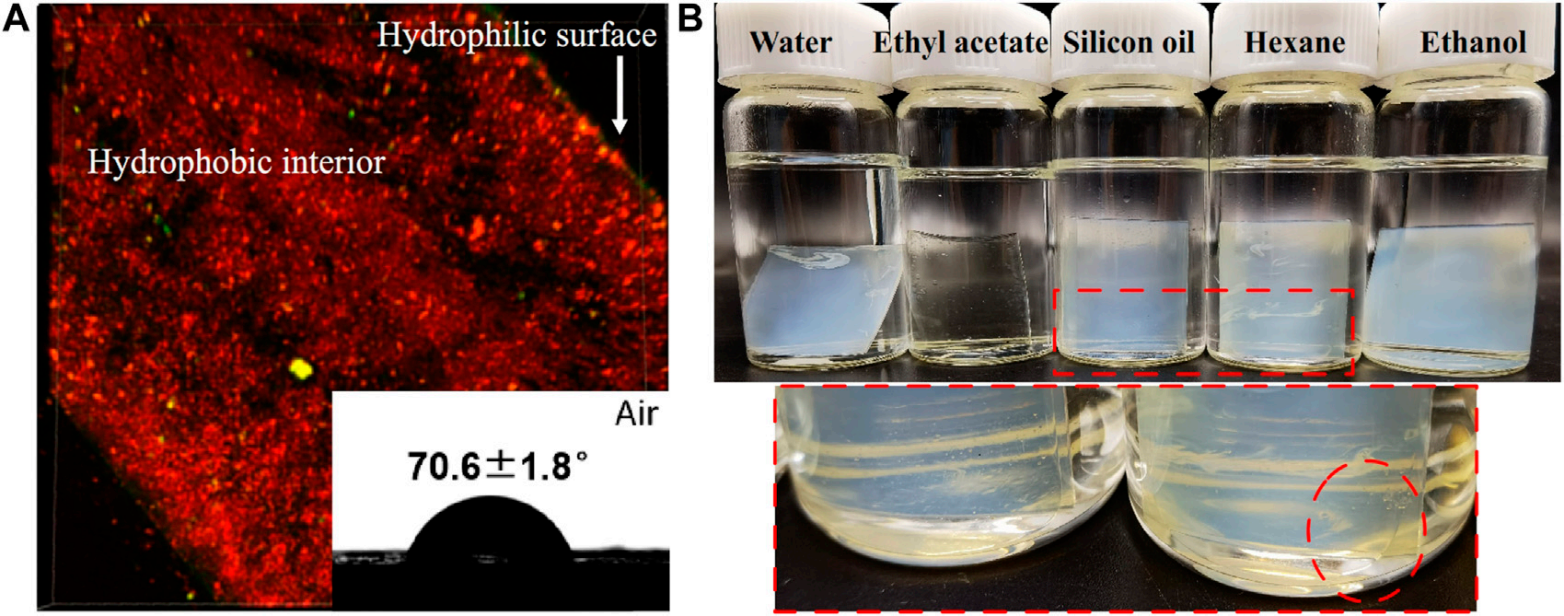
The wettability and solvent displacement test of PMAA/PEI gel. **(A)** The cross section of gel dyed by sodium fluorescein and DiI is mainly oleophilic but shows hydrophilic on the surface. The contact angle of water on the flat surface reaches 70.6 ± 1.8°; **(B)** The photo of PMAA/PEI gel soaked in water, ethyl acetate, silicone oil, n-hexane and ethanol for 72 h show that it becomes transparent when soaked in ethyl acetate, while part of the water in the gel soaked in silicone oil and n-hexane can be replaced by organic solvents.

PMAA/PEI gel has very obvious hydrophobicity. As shown in Figure 4A, when the gel is dyed with hydrophilic dyes (sodium fluorescein, yellow fluorescence) and hydrophobic dyes (DiI, red fluorescence) respectively, it can be observed under the laser confocal microscope that the gel section is almost completely red. So, it is dyed by hydrophobic dyes. However, the surface of PMAA/PEI gel can be dyed by hydrophilic dyes, indicating that there is a hydrophilic layer on the surface of gel. Supplementary Figure S2 shows the contact angle of PMAA/PEI gel in air and the contact angle of silicone oil under water. The contact angles of silicone oil on the surface and inside of gel show obvious differences, which are 138.4 ± 7.0° and 115.5 ± 8.1°, respectively. This shows that the interior of gel has stronger oleophilicity. This is because the incompletely assembled polymer chains on the gel surface which can make the gel surface more hydrophilic. The interior of gel cannot be observed because it is opaque. The illustration in Figure 4A shows the water contact angle of PMAA/PEI gel in the air, which has reached more than 70°, exceeding the hydrophobic/hydrophilic limit (Vogler, 1998; Tian and Jiang, 2013). Therefore, it can be considered that the surface of PMAA/PEI gel is hydrophobic. During the preparation of gel, its surface and interior were not treated differently, so it can be inferred that the interior of gel is also hydrophobic. However, the internal network of PMAA/PEI gel actually contains a lot of water (about 65%), and the two polymers that make up the gel are also hydrophilic, which indicates that the two polymer networks inside the gel have strong assembly ability and can completely wrap water. In Figure 4B, we carried out the solvent displacement experiment of gel. The experiment confirmed that the water in PMAA/PEI gel can be replaced by some organic solvents, such as ethyl acetate, silicone oil, n-hexane, etc. For ethyl acetate, it can even obviously enter the gel network, and make the gel transparent. The above experiments confirmed that there are a large number of hydrophobic networks in the gel, which has better affinity for many organic solvents than water.

### Structure of hydrogel

**FIGURE 5:**
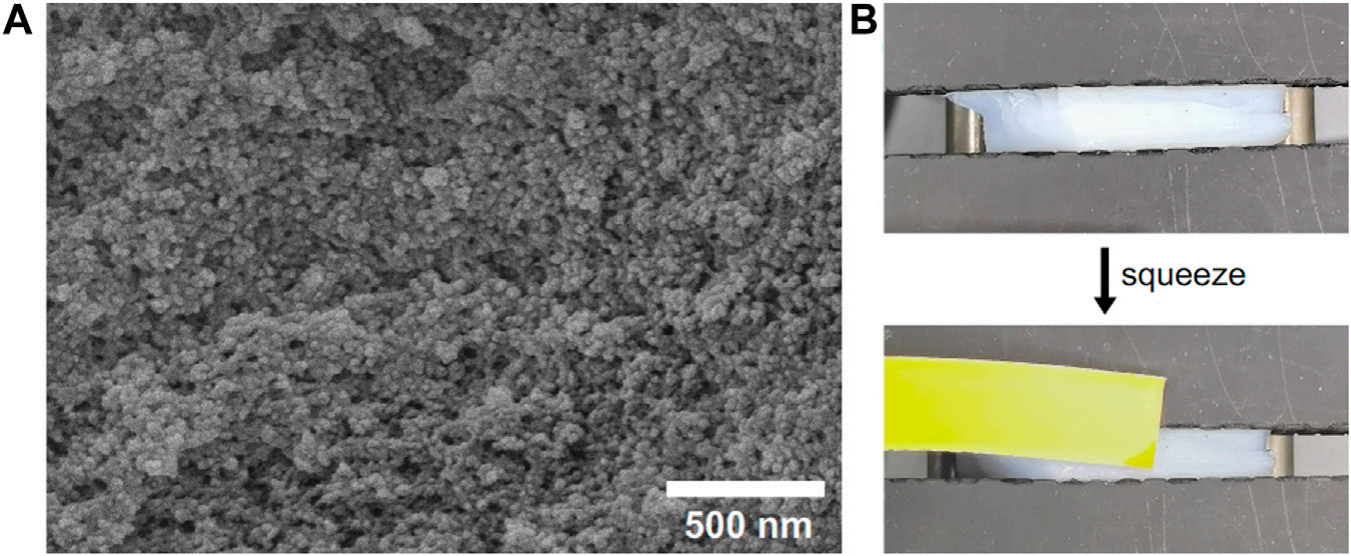
PMAA/PEI gel has a porous structure and can extrude water. **(A)** Porous structure of gel; **(B)** Under the action of external force, the water in the pore structure of gel can be squeezed out.

As a hydrophobic PMAA/PEI gel containing a lot of water, there is an obvious phase separation structure in it. Figure 5A shows that PMAA/PEI gel material has a nano-sized porous structure. This shows that the polymer in the gel has been assembled into a relatively rigid hydrophobic network, while the original water is filled in the porous structure formed by nano-sized phase separation. In addition, a large number of water filled porous structures in PMAA/PEI gel can also be deformed under external force extrusion, and the water filled in the porous structure will be partially squeezed, as shown in Figure 5B and Supplementary Video S2. Since the gel used in the test is a 1:1 M ratio of PEI and PMAA, it can be completely assembled without residue in water. The inspection result of the pH test paper used to test the pH of the extruded water in the experiment is close to pH = 7, which also proves that there is almost no excess PEI or PMAA residue in the extruded water, that is, the water in the pores is almost pure water.

### Interactions in hydrogel

**FIGURE 6:**
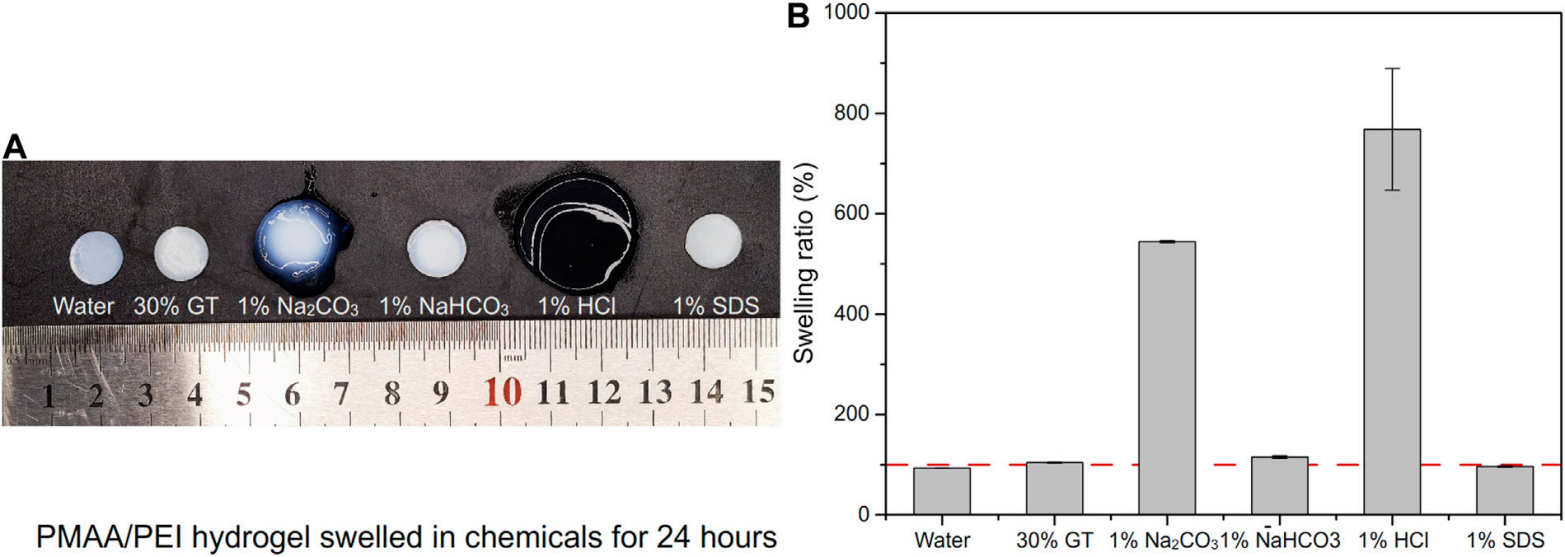
The swelling of PMAA/PEI gel in several solutions. **(A)** Photos of PMAA/PEI gel after swelling in water, 30% GT, 1% sodium carbonate, 1% sodium bicarbonate, 1% hydrochloric acid and 1% SDS solution for 24 h. The swelling is very obvious in hydrochloric acid and sodium carbonate solution; **(B)** Swelling rate data in several solutions. The gel swells severely in hydrochloric acid and sodium carbonate solutions, shrinks slightly in water and SDS, and swells slightly in 30% GT and sodium bicarbonate solutions.

PMAA/PEI gel, as a hydrogel that does not consist chemical crosslinkers but only relies on hydrogen bonds, ionic bonds and hydrophobic interactions, has shown strong resistance to a variety of destructive agents. As shown in Figure 6A, PMAA/PEI gel shows different swelling states after soaking in water, 30% GT, 1% sodium carbonate, 1% sodium bicarbonate, 1% hydrochloric acid and 1% SDS solution for 24 h. Figure 6B shows the swelling degree of PMAA/PEI gel in various solvents. The gel in water showed a slight shrinkage, which was due to the tight hydrophobic network in the gel. However, in 30% GT solution, gel has almost no swelling. This is because PMAA/PEI gel is assembled by both ionic bonds and hydrogen bonds, and the ionic bonds are difficult to be destroyed by GT, so the swelling of gel is weak. In 1% sodium carbonate solution, the pH of the sodium carbonate solution can reach 10, which will seriously reduce the charge of PEI. The negative charge of PMAA network will also increase, leading to the destruction of the ionic bond assembly of gel, so the gel has a very obvious swelling. However, the pH of 1% sodium bicarbonate solution is only 8. At this pH value, the charge of PEI is less affected, so gel only shows weak swelling. In 1% hydrochloric acid, the pH has reached about 1. The negative charge of the PMAA network is basically neutralized, while only the PEI network remains positively charged. Therefore, the gel shows very serious swelling and has become transparent, indicating that the assembly structure of PEI/PMAA has been destroyed, and the phase separation and hydrophobic interaction have basically disappeared. The gel soaked in SDS solution did not appear to have obvious changes in appearance, but SDS was able to participate in the assembly of hydrophobic structure, showing a very obvious difference in the subsequent mechanical property test.

### Mechanical properties

**FIGURE 7:**
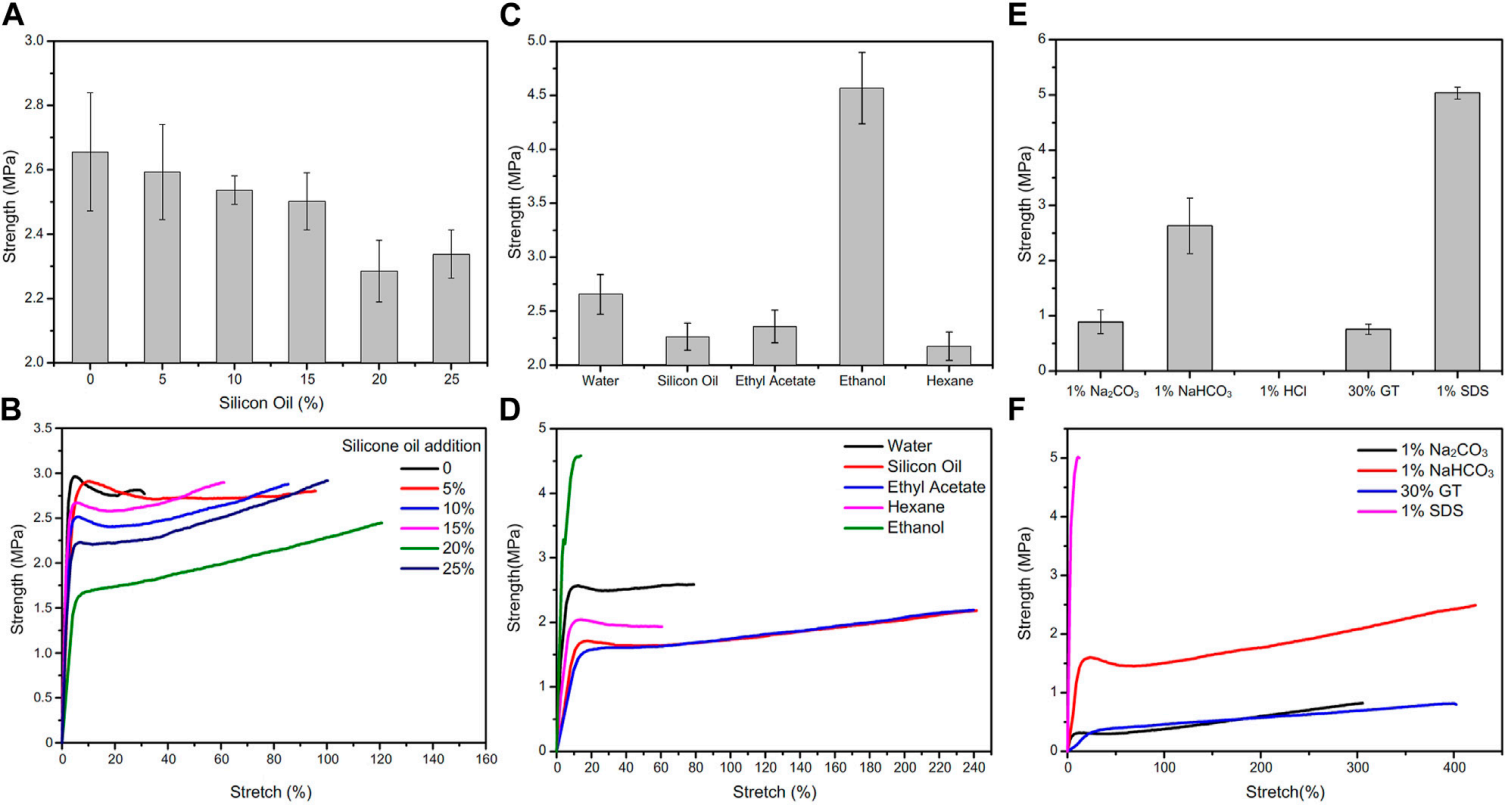
Change of tensile strength of PMAA/PEI gel under different conditions. **(A)** Adding silicone oil of different content to PMAA/PEI gel, the strength of gel slightly decreased with the increase of silicone oil content; **(B)** For the tensile force curve of gel with different silicone oil content, the breaking elongation of gel increases with the increase of silicone oil content; **(C)** The tensile strength of gel soaked in water and several organic solvents for 24 h; **(D)** Gel force curve after immersion in water and several organic solvents. The elongation at break of gel soaked in ethyl acetate and silicone oil increased a lot, while the modulus of gel soaked in ethanol increased a lot; **(E)** Tensile strength of PMAA/PEI gel soaked in several solutions; **(F)** The tensile force curve of gel soaked in several solutions.

PMAA/PEI gel shows high mechanical strength and tolerance to various environments. As shown in Figure 7A, after the silicone oil dispersed in the gel up to 25% (compared with the mass ratio of the polymers in the gel), the tensile strength of the gel only decreased slightly. On the one hand, PEI and MAA are amphiphilic and can effectively disperse silicone oil that is not miscible with water; On the other hand, there are a large number of hydrophobic pore structures in gel, and these dispersed silicone oil can be directly filled in the hydrophobic pore structure of gel, without affecting the main force network structure of gel, and without defects due to the addition of these silicone oil. Figure 7B shows the force curve of these gel stretching to fracture, and the elongation at break of the gel added with silicone oil has increased significantly. This shows that some silicone oils participate in the hydrophobic interaction in the gel network structure. When stretching, these silicone oils act as lubricants, so the elongation of gel increases. In addition, there are very obvious necking points in these force curves. This means that near the necking point, the weak hydrophobic interaction force in the gel network is destroyed by external force, and the sliding between hydrophobic chains makes the stress drop.

PMAA/PEI gel can also show the same mechanical property changes as silicone oil after solvent replacement in several organic solvents. The strength after solvent replacement is shown in Figure 7C, and the force curve is shown in Figure 7D

. The tensile strength of PMAA/PEI gel soaked in ethyl acetate, n-hexane and silicone oil for 24 h decreased compared with that soaked in water, but the elongation at break of gel soaked in silicone oil and ethyl acetate increased significantly. This indicates that these solvents have partially replaced the water in the gel and entered the hydrophobic pores in the gel to play a certain role in lubrication. However, the strength of gel soaked in ethanol was significantly improved, and the elongation at break was significantly reduced, while the modulus was increased. This is because ethanol can be miscible with water, so ethanol can simultaneously enter the hydrogen bond assembly structure and hydrophobic pores in the gel. However, the polarity of ethanol is lower than that of water, and the ability to form hydrogen bonds is also weaker than that of water. Therefore, after soaking in ethanol, the ionic bond assembly of gel is strengthened. Then, part of the bound water in the assembly structure is replaced, making the assembly structure more compact. Therefore, the mechanical properties of gel become stronger and the modulus increases.

After soaking in acid, alkali, SDS and guanidine thiocyanate solutions, the mechanical properties of PMAA/PEI gel also show obvious changes. The strength changes are shown in Figure 7E, and the force curve is shown in Figure 7F. The first is the PMAA/PEI gel soaked in hydrochloric acid. Due to the very serious swelling, its own strength is significantly reduced and even difficult to measure. The strength of PMAA/PEI gel soaked in 1% sodium carbonate solution also decreased significantly. The strength of gel samples soaked in sodium bicarbonate solution has no obvious change, because the alkalinity of sodium bicarbonate solution is weak, which has little effect on the electrification of gel. In addition, the gel soaked in 30% GT solution showed no obvious swelling and transparency changes, but its strength also decreased significantly. This is due to the destruction of hydrogen bond by GT, and the electrostatic interaction that is less affected plays a role in maintaining the structure and partial strength of gel. The strength of gel samples soaked in 1% SDS solution changes significantly, because SDS is not only charged, but also has hydrophilic and hydrophobic structures, so it can participate in both hydrophobic and electrostatic assembly in PMAA/PEI gel. In this process, part of the water in the gel structure will be replaced by SDS. Because the molecule of SDS is much larger than that of water, the entry of SDS will fill more gel pores, making PMAA/PEI gel more compact, resulting in a significant increase in the strength and modulus of gel.

### Under water and organic solvents adhesive and applications

**FIGURE 8:**
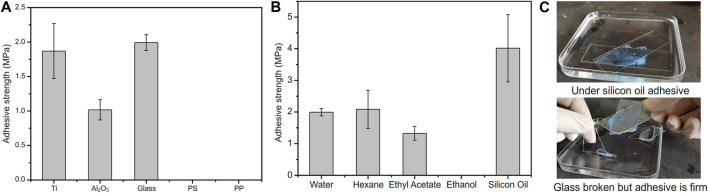
Adhesion performance of PMAA/PEI adhesive in water and organic solvent. **(A)** PMAA/PEI adhesive can firmly adhere titanium, aluminum oxide, glass and other polar materials under water, but shows almost no adhesion strength to non-polar materials such as PS and PP; **(B)** PMAA/PEI adhesive can achieve high strength adhesion to glass in a variety of organic solvents, such as n-hexane, ethyl acetate and silicone oil, but it cannot achieve adhesion in ethanol. The strength of adhesion glass under silicone oil is even higher than that under water; **(C)** The adhesion process of PMAA/PEI adhesive to glass under silicone oil. After the polymerization of PMAA/PEI adhesive, the PMAA/PEI adhesive cannot be damaged even when the glass is damaged under the action of tangential force.

PMAA/PEI gel, as a bionic barnacle glue material, has a variety of complex interactions similar to barnacle glue, such as hydrogen bond, ionic bond, hydrophilic hydrophobic phase transition, hydrophobic interaction, and can also realize rapid thermal polymerization and curing at room temperature (about 180 s). Therefore, it has great potential as a bionic adhesive material. Figure 8A shows the underwater adhesion strength of PMAA/PEI gel to a variety of materials. It has very high underwater adhesion strength to polar materials such as titanium sheet, aluminum oxide, glass, and even far exceeds the underwater adhesion strength of barnacle glue, but it has almost no adhesion strength to non-polar materials such as PS and PP. This is because the components of PMAA/PEI gel are almost completely hydrophilic before polymerization, and hydrophobic interaction can be formed only after assembly after polymerization, which makes it difficult to generate strong interaction between the gel network and non-polar materials before polymerization, while after polymerization, the gel network has been assembled, movement is limited, and it is also difficult to generate sufficient interface interaction with non-polar surfaces. For the surface of polar materials, before gel polymerization, a large amount of electrostatic and hydrogen bonding interactions is enough to make the PEI chain that forms the gel network and MAA monomer adsorb and replace part of the bound water on the surface of polar materials, and the interaction of these interfaces is not affected after polymerization. The replaced interface water can be directly filled in the pores of gel without interfering with the interface interaction and the polymerization process of gel. PMAA/PEI gel has more electricity and more hydrogen bonds than barnacle glue. Therefore, the underwater adhesion strength of PMAA/PEI gel to polar material surface is better than barnacle glue.

For many adhesives, it is a considerable challenge to achieve adhesion in the presence of oil with low surface tension and high viscosity (such as silicone oil), because low surface tension means that these oils can easily wet various surfaces and are difficult to be rapidly replaced at the interface, resulting in the difficulty of achieving effective interface interaction. Previous experiments have confirmed that PMAA/PEI gel has a porous structure and can wrap water or a variety of organic solvents without affecting its strength. Therefore, we believe that even the silicone oil on the interface can be effectively replaced by PMAA/PEI gel adhesive and filled in the pores of gel without having an obvious impact on the adhesion process. Figure 8B shows the adhesion strength of PMAA/PEI gel to glass in water and various organic solvents. The hydrophobic self-assembly inside the gel is affected in ethanol, which makes it difficult for the gel to cure, the adhesion strength of PMAA/PEI gel in low polar solvents is almost no lower or even higher than that under water, especially under silicone oil, which even exceeds that under water, and even approaches the strength of gel itself. This shows that PMAA/PEI gel can quickly replace the oil layer at the interface and achieve effective interface interaction. Compared with underwater adhesion, PMAA/PEI pre-gel solution will not be diluted by water, so its bulk strength is higher. As shown in Figure 8C, the photo shows the process of glass sheet adhesion under silicone oil, and the corresponding Supplementary Video S3 is also included in the Supplementary Material. After curing (about 180 s), PMAA/PEI gel under silicone oil adhesive has firmly stuck two pieces of glass under silicone oil together, and even if external force is used to destroy the glass, it will preferentially destroy the glass rather than the adhesion position. This shows that PMAA/PEI, as a new adhesive of bio-inspired barnacle glue, has very good adaptability in underwater and oil environments.

## Conclusion

In this work, we designed a gel composed of PMAA/PEI by imitating the adhesion principle of barnacle glue. The gel was assembled internally by hydrogen bonding, electrostatic force and hydrophobic interaction between PMAA/PEI assemblies, without chemical crosslinkers. The gel can achieve rapid curing in only 180 s at room temperature, and has very high mechanical properties. Its tensile strength reaches 2.66 ± 0.18 MPa. The gel has a porous phase separation structure, and the pores can be partially replaced by some organic solvents. Similar to barnacle glue, PMAA/PEI gel can achieve high strength underwater and organic solvents adhesion to a variety of polar materials, such as underwater adhesion strength of 1.99 ± 0.11 MPa to glass, the adhesion strength under silicone oil can even reach 2.70 ± 0.21 MPa. This is because PMAA/PEI assembly can form hydrogen bond, electrostatic force and other interactions at the interface, and can also transfer the water or organic solvent in the interface layer to the pores in the gel without affecting the strength of the gel itself. This new bio-inspired gel can not only rely on its own high mechanical properties and porous structure as a new bionic material, but also can be used as a new bionic adhesive for adhesion in a variety of organic solvents, and provides a new design idea for the new adhesive.

## Data Availability

The original contributions presented in the study are included in the article/Supplementary Material, further inquiries can be directed to the corresponding authors.
