# Supplementary material for: Barnacle inspired high-strength hydrogel for adhesive
Source: Front Bioeng Biotechnol. 2023 Apr 3;11:1183799. doi: 10.3389/fbioe.2023.1183799 (PMC10106642; doi:10.3389/fbioe.2023.1183799)
Supplement: Supplementary file 4 [file DataSheet1.docx]

Supplementary Material

Barnacle inspired high-strength hydrogel for adhesive

**Dezhao Hao^1,2^, Xingchao Li^1,2^, Enfeng Yang^1,2^, Ye Tian^1,2,3*^, Lei Jiang^1,2^**

*** Correspondence:** Ye Tian: tianyely@iccas.ac.cn

# Supplementary Figures and Tables

## Supplementary Table

| PMAA/PEI molar ratio | Mass of 50% PEI solution (g) | Mass of water (g) | Mass of MAA (g) | Mass of APS (g) | Water content (wt%) |
| --- | --- | --- | --- | --- | --- |
| 0.5 | 30 | 41.67 | 15 | 0.15 | 65.38 |
| 0.75 | 30 | 55.83 | 22.5 | 0.225 | 65.38 |
| 1 | 30 | 70.00 | 30 | 0.3 | 65.38 |
| 1.25 | 30 | 84.17 | 37.5 | 0.375 | 65.38 |
| 1.5 | 30 | 98.33 | 45 | 0.45 | 65.38 |

## Supplementary Table 1. Different proportion of PMAA/PEI pre-gel solution Under different PMAA/PEI ratios. The amount of water added in the pre-gel solution is adjusted to ensure that the water content of these gels is the same.

## Supplementary Figure


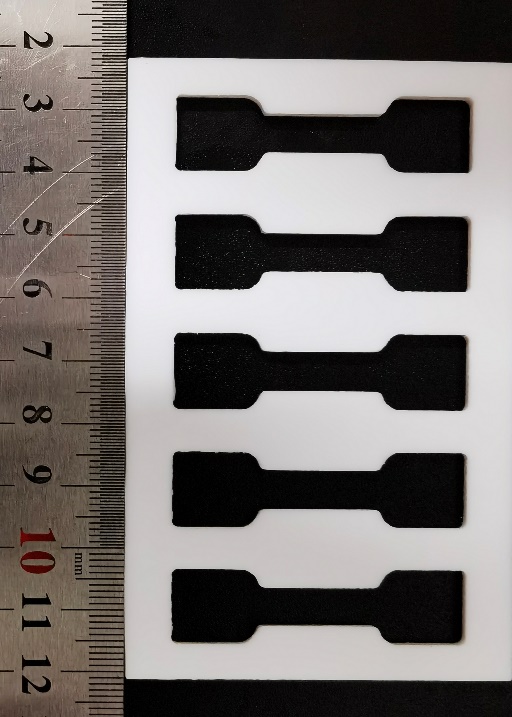


**Supplementary Figure 1.** The mold for mechanical properties test. The material is polytetrafluoroethylene, the thickness is 2 mm, and the width of the tensile part is 6 mm.


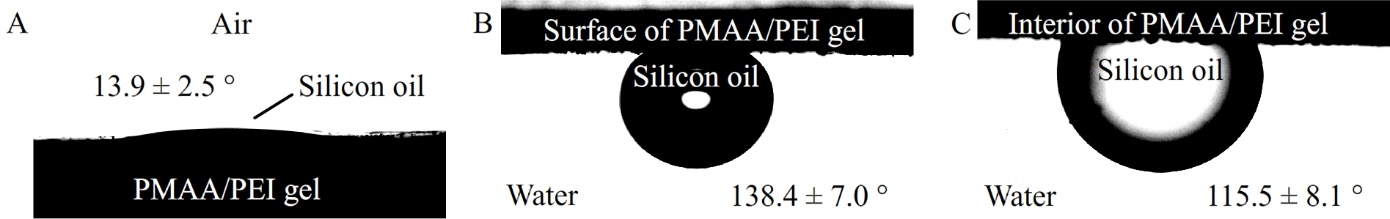


**Supplementary Figure 2.** Contact angle of PMAA/PEI gel under different conditions. (A) Contact angle of silicone oil in air is 13.9 ± 2.5 °; (B) Underwater contact angle of silicone oil on the surface of the gel is 138.4 ± 7.0 °; (C) Underwater contact angle of silicone oil on the inside of the gel is 115.5 ± 8.1 °.
